# Time-related trends in variability of cIMT changes in statin trials

**DOI:** 10.1016/j.dib.2015.12.029

**Published:** 2015-12-30

**Authors:** Michael H. Davidson, Joanne E. Tomassini, Erin Jensen, David Neff, Adam B. Polis, Andrew M. Tershakovec

**Affiliations:** a Preventive Cardiology, The University of Chicago Pritzker School of Medicine, 515 North State Street Suite 2700, Chicago, IL 60610, USA; b Global Clinical Development, Merck & Co., Inc., 2000 Galloping Hill Road, Kenilworth, NJ, 07033, USA

## Abstract

This brief article provides complementary data supporting the results reported in “Changing Characteristics of Statin-related cIMT Trials from 1988 to 2006” [1]. That article described time-related trends in baseline factors and study characteristics that may have influenced the variability of carotid intima media thickness (cIMT) endpoints (mean of mean and maximum common carotid artery [CCA]/cIMT) in published statin trials. In this brief report, additional details for the studies included in the analysis, and further supporting data, including mean of the maximum CCA/cIMT changes and subgroup data (mean and maximum CCA/cIMT) are provided. For the analysis, study-level data was extracted from 17 statin cIMT trials conducted during 1988–2006, selected on the basis of having at least one statin monotherapy arm in the absence of mixed therapy, and baseline- and study-end values for mean mean and mean maximum CCA/cIMT endpoints. The baseline mean CCA/cIMT, maximum mean CCA/cIMT and LDL-C levels, and annualized cIMT changes were estimated for the overall studies, those conducted before/after 2000, and in risk-based subgroups. Interestingly, all 8 studies conducted before 2000 were significant for cIMT change in which patients did not receive prior LLT; whereas after 2000, the results were more variable and in 4 of 6 trials that did not show a significant cIMT change, patients had received prior treatment. Baseline mean maximum cIMT and LDL-C levels, and annualized changes in studies conducted before 2000 were higher than those conducted after 2000, similar to the results reported in the original article for the mean mean cIMT endpoint. These findings were consistent across study populations of patients with CHD risk versus those without, and in studies with greater LDL-C reductions and with thickened baseline cIMT at study entry for both mean and maximum cIMT changes. Taken together, these results are consistent with trends in recent years toward greater use of lipid-lowering therapy and control of LDL-C that may have impacted the variability in the results of cIMT studies.

**Specifications table**

**Table:** 

Subject area	*Vascular Biology*
More specific subject area	*Carotid artery imaging*
Type of data	*Tables and figures*
How data was acquired	*Study-level data from published statin cIMT trials*
Data format	*Aggregate study-level data*
Experimental factors	*Brief description of any pretreatment of samples*
Experimental features	*Very brief experimental description*
Data source location	*Published cIMT trials as referenced*
Data accessibility	*Data are supplied with this article*

**Value of the data**

• The relationship between baseline and study characteristics and the variability of cIMT change in statin cIMT trials over time has not been well-studied.
• These data show time-related trends in baseline and study characteristics that may influence the variability of mean mean and mean max cIMT results in statin clinical trials.
• Statin cIMT trials conducted before 2000 had greater cIMT baseline values, LDL-C reduction and annualized cIMT changes than studies after 2000.
• These trends are consistent with increased statin treatment and management of LDL-C lowering over recent years, bringing into question the utility of cIMT as a surrogate marker in clinical trials given the high standard of background statin therapy.
• These results highlight the need to consider such factors in the design of future cIMT trials.

## 1 Data

The predictive power of cIMT as a surrogate marker for the assessment of CVD risk reduction in lipid-lowering trials may be limited in some settings due to differences in study design, cIMT methods, and patient characteristics [2], [3], [4]. With increased use of lipid-lowering therapy over recent years, it is possible that patient characteristics and study designs of cIMT trials have also changed in a manner which could influence the utility of cIMT in assessing LLT treatment effects. This analysis was undertaken to better understand how baseline factors and study characteristics may influence the variability of cIMT changes in intervention trials.

A summary of the cIMT trials included in the analyses reported in the original article [1] and in this brief report are summarized in Table 1. All 8 of the studies that were conducted before year 2000 were significantly positive for cIMT change, and the patients in those trials did not receive prior lipid-lowering therapy. However, the 9 studies conducted after 2000 were more variable for cIMT changes with 6 of these not showing significant cIMT change, the majority of which (4 of 6 trials) included patients that had received prior lipid-lowering therapy. The baseline mean mean and mean maximum cIMT and LDL-C levels were also generally greater for those studies conducted before 2000 than after 2000. The durations of the trials were also longer (≥2 years) in those conducted before 2000.

Table 2 summarizes the mean maximum baseline CCA/cIMT level and annualized changes for the overall studies (1988–2006) and those conducted before and after 2000, as well as for various study populations. For the overall studies combined (1988–2006), the baseline mean maximum CCA/cIMT level was 1.0866 mm and the annualized rate of change for the mean maximum CCA/cIMT from baseline was −0.0023 mm/yr (95% CI: −0.0050, −0004), indicating a statistically significant annualized change in cIMT. Baseline mean maximum cIMT levels were higher in populations of studies conducted before 2000 (1.2550 mm) than after 2000 (1.0181 mm), as well as in study populations of patients with CHD risk versus those without, and in those studies with greater LDL-C reductions, and those with thickened baseline cIMT. Annualized rates of change in maximum CCA/cIMT levels were also larger for the combined study populations conducted before year 2000 (−0.0134 mm/yr) than after 2000 (−0.0083 mm/yr), and for those studies in which patients had thickened baseline cIMT at study entry and also those which showed greater LDL-C reductions.

The baseline mean CCA/cIMT and annualized changes observed in pooled studies conducted before and after 2000 for various prespecified subgroups of interest are displayed in Table 3. Consistent with the findings in the overall analysis cohort, baseline cIMT levels and annualized change for mean CCA/cIMT were generally greater in all subgroups assessed for the combined studies conducted before 2000 than after 2000. Similarly, mean max CCA/cIMT and annualized change in studies were also generally greater in all subgroups assessed in studies conducted before 2000 than after 2000 (Table 4).

## 2 Experimental design, materials and methods

### 2.1 Study design

**Fig. 1:**
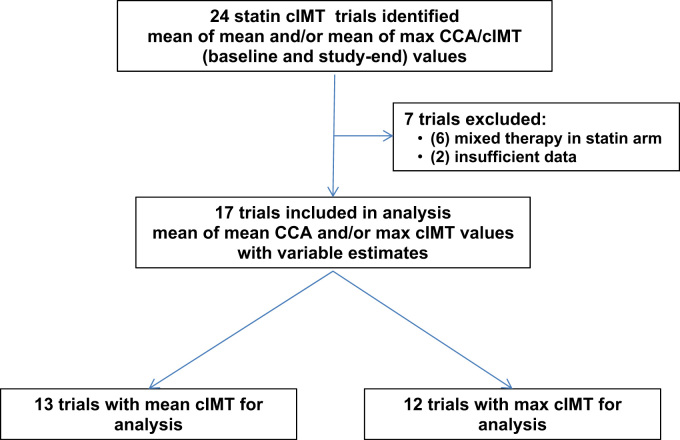
Study selection flow chart.

This was an exploratory analysis of study-level data from statin treatment arms of published cIMT imaging trials, as previously described in the original study article [1]. Fig. 1 displays the method of trial selection for the analysis. Following a detailed review of cIMT trials in the literature, 24 statin trials were identified that had both baseline- and study-end measurements for mean of the mean CCA/cIMT (mean of all mean measurements on CCA or a single mean cIMT value when not available), and/or mean of the maximum CCA/cIMT (mean of all maximum mean measurements on CCA, or single maximum mean or bulb values when not available). Of these, 7 trials were excluded due to insufficient data or mixed lipid-lowering therapy in the statin arm, and 17 studies conducted during 1988–2006 were selected on the basis of having at least one statin monotherapy arm in the absence of mixed therapy, baseline- and study-end values for mean of the mean and mean of the maximum CCA/cIMT. The selected studies included a mix of placebo (ACAPS, ARBITER II, BCAPS, CAIUS, CAPTIVATE, CASHMERE, KAPS, LIPID, METEOR, PLAC II, REGRESS) and active-controlled (ARBITER I, ASAP, RADIANCE I, RADIANCE II, ENHANCE, VYCTOR) studies (Table 1 and Fig. 1) [5], [6], [7], [8], [9], [10], [11], [12], [13], [14], [15], [16], [17], [18], [19], [20], [21]. There were 13 trials with mean mean CCA/cIMT as the primary endpoint and 12 with mean maximum CCA/cIMT endpoints; these were assessed in 2 separate, but similar analyses.

### 2.2 Data extraction and analysis

Only aggregate data in the published literature were collected from the studies. Demographic information (e.g., age and gender), study characteristics (study dates, CHD risk, heterozygous familial hypercholesterolemia [FH], baseline LDL-C, HDL-C and TG levels), and baseline- and study-end measurements for mean mean CCA/cIMT and mean max CCA/cIMT, and variability estimates were extracted from the publications. Baseline means of the mean CCA/cIMT and maximum CCA/cIMT levels were summarized for the overall population of studies, those conducted before and after 2000 (the median of enrollment dates for all studies), and studies that enrolled patients based on CHD risk, heterozygous FH, and thickened baseline cIMT. Subgroups of patients categorized by LDL-C reduction, age and gender were also assessed. Dichotomized variables for the subgroups were prespecified based on median values for the combined studies.

Studies were combined using a meta-analytic approach. The 13 trials included in the mean mean CCA/cIMT analysis were: ARBITER I, REGRESS, ASAP, KAPS, LIPID, METEOR, BCAPS, CASHMERE, CAPTIVATE, ENHANCE (simvastatin), ARBITER II (placebo), and Radiance I & II (atorvastatin). The 12 studies included in the analysis of mean maximum CCA/cIMT were: ACAPS, ARBITER I, BCAPS, CAIUS, CAPTIVATE, ENHANCE (simvastatin), REGRESS, METEOR, PLAC II, VYCTOR, and RADIANCE I & II (atorvastatin).

Study-end values were reported as a change from baseline in primary variables (mean mean or mean max CCA/cIMT) indicating regression and/or progression of cIMT. Annualized rates of change (regression and/or progression) were extracted as treatment differences for placebo-controlled studies and as change from baseline in each individual treatment arm for active-control studies. For active-controlled studies with 2 or more monotherapy arms, each treatment arm was included in the analysis as a separate data point. For trials in which annualized rates of change was not provided, it was calculated by dividing the reported change in cIMT by the duration of the study in years.

Estimates of variance for the baseline and study-end values for mean mean and mean max cIMT were extracted from the publications. In most cases the standard deviation was reported for baseline values and the standard error (SE) was reported for study-end values. In some instances the SE was not reported, and it was calculated from the reported 95% CIs or *p*-values for the treatment difference (placebo-controlled) or change from baseline (monotherapy arms) in cIMT. The rate of cIMT change was standardized (annualized) for this analysis; however, the SE reported in the publication was used regardless of study duration. An overall pooled estimate for the baseline CCA/ cIMT value and annualized rate of cIMT change was calculated by weighting each individual study by the inverse of the square of its standard error which was extracted from the publications. This resulted in studies with small variability having more weight in the pooled values than studies with large variability. Overall pooled estimates for the baseline CCA/cIMT value and annualized rate of change for mean of mean CCA and mean of max cIMT, were calculated using a fixed-effects model, by weighting each individual study by the inverse of the square of its standard error; thus, studies with small variability had more weight in the pooled values than studies with large variability.

## Source of funding

Merck Sharp & Dohme Corp., a subsidiary of Merck & Co., Inc.

## Disclosures

Dr. Davidson received research support from Abbott Labs, AstraZeneca, Merck and Roche, has served on speaker׳s bureaus for Abbott Labs, AstraZeneca, GSK, MSP and Takeda and as a consultant/advisory board member for Abbott Labs, Aegerion, Amgen, AZ, Daiichi-Sankyo, DTC MD, Esperion, GSK, iMD, Kinemed, LipoScience, Merck, MSP, NovoNordisk, Omthera, Professional Evaluation Inc., Roche, Sanofi-Aventis, Synarc, Takeda, and Vindico, and holds Equity/Brd of Directors for Omthera, Professional Evaluation, Inc. Medical Company (Brd Dir), and Sonogene (Brd Dir). Drs. Neff, Tershakovec, and Tomassini, and Mr. Polis are employees of Merck Sharp & Dohme Corp. a subsidiary of Merck & Co., Inc., Kenilworth, NJ, and may hold stock/stock options in the company. Ms. Jensen is a contract employee of Merck.

## Figures and Tables

**Table 1:** Summary of the included trials.

**Study (Reference)**		**Treatment (mg)**⁎⁎⁎		**Age (yr)**	**Female (%)**	**Population**	**Prior treatment**	**Study length (yr)**	**Study (yr)**	**Baseline IMT thickness (mm)**		**Baseline mg/dl (mmol/l)**		
**Arm**	***n***	**Mean CCA/cIMT**	**Mean max CCA/cIMT**	**LDL-C**	**HDL-C**	**TG**
**Statin studies**														
ACAPS⁎⁎	**L20-40**		231	62	49	Asymptomatic CAD; LDL-C 60–90th percentiles; IMT≥1.5 - <3.5 mm	No prior LLT for 1 yr	3	1989–1990	*L***±***W*=1.15	*L***±***W*=1.33	155	45.8 (1.19)	140 (1.58)
[9]	Pbo=1.14
Pbo=1.32	(4.01)
Pbo		230
ARBITER I⁎⁎	**P40**		70	60	29	Adults (>18 yr), ATPII lipid-lowering criteria	No prior LLT	1	1999–2001	*P*=0.615 *A*=0.625	*P*=0.808 *A*=0.935	152	49	207 (2.34)
**A80**		68	(3.94)	(1.27)
[20]

ASAP⁎⁎	**A80**		160 163	48	61	hFH;	Untreated or treated ≤1 yr, LDL-C >173 mg/dL (4.48 mmol/l)	2	1997–1998	*A*=0.86 *S*=0.87	NA	315 (8.16)	46 (1.19)	165 (1.86)
[19]	**S40**		mean CCA ≥0.7 mm and/or ≥0.9 mm in bulb

BCAPS⁎⁎	Met		200	61.8	54.5	Asymptomatic plaque (focal IMT >1.2 mm) in right coronary artery	No beta blocker or statin	3	1991–1994	Met=0.92 *F*=0.886	Bulb:	158 (4.09)	53.2 (1.38)	104 (1.18)
[10]	Met=1.936 *F*=1.893
	Pbo=0.898
**F40**		200
Pbo=1.875
Pbo		200
CAIUS⁎⁎	**P40**		151	55	47	Moderate HC, LDL-C 150–250 mg/dL (3.88–6.47 mmol/l), ≥1 cIMT lesion 1.3–3.5 mm	No prior LLT	≤3 yr	1991–1995	NA	*P*=1.06	181 (4.69)	52.5 (1.36)	138 (1.56)
[15]	Pbo=1.04
Pbo		154	
CAPTIVATE⁎	Pact 100		443	55	39	hFH, LDL-C >100 mg/dL (>2.6 mmol/l); max cIMT>0.7 -≤2.5 mm	Statin Rx, 〈or〉 24 mon	1	2004–2005	Pac=0.785 Pbo=0.775	Pac=0.937 Pbo=0.927	140 (3.63)	52 (1.35)	130 (1.47)
[16]	**Pbo**		438	
CASHMERE⁎	**A80**		192	57	100	Postmenopause women ≤70 yr; LDL-C 130–190 mg/dL (3.36–4.86 mmol/l)	No statins >3 mon w/in last yr, or no LLT w/in last 6 wks	1	2003–2006	*A*=0.699	NA	159 (4.12)	66.6 (1.72)	120 (1.36)
[17]	Pbo		206		Pbo=0.683
KAPS⁎⁎	**P40**		212	57	0	LDL-C 164 mg/dL (≥4.25 mmol/l), BMI ≤32 kg/m^2^	No prior LLT	3	1989–1990	1.35 (overall)	NA	189 (4.90)	46 (1.19)	150 (1.70)
[18]	Pbo		212	
LIPID⁎⁎	**P40**		273	61	12	MI or hospitalization for unstable angina†	2 mon run-in diet, no prior LLT	4	1990–1992	*P*=0.804 Pbo=0.786	NA	154 (3.99)	34.7 (0.90)	151 (1.71)
[13]
Pbo		249	
METEOR⁎⁎	**R40**		702	61	12	Asympotmatic, moderately elevated cholesterol with low CVD risk per ATPIII‡	No LLT 12 mon prior	2	2002–2004	*R*=0.76	*R*=1.15 Pbo=1.17	165 (4.27)	50 (1.30)	130 (1.47)
Pbo=0.76
[7]

Pbo		282
PLAC II⁎⁎	**P20→40**^a^		75	61	12	CAD, LDL-C 60–90th percentile, ≥1 IMT lesion ≥1.3 mm	No prior LLT	3	1988–1990	*P*=1.01	*P*=1.32 Pbo=1.32	166 (4.30)	41 (1.06)	171 (1.93)
[6]	Pbo=1.01
Pbo		76
RADIANCE I⁎	**A10, 20, 40, 80** A+T60		454	61	12	hFH	Aspirin 30%; beta blocker 20%; ACE inh or ARB 16–19%; E 21%	2	2003–2004	*A*=0.72 *A*+*T*=0.71	*A*=1.15 *A*+*T*=1.09	139 (3.60)	53 (1.37)	97 (1.10)
[11]	450
RADIANCE II⁎	**A10, 20, 40, 80** A+T60		375	57	37	Mixed HL eligible for statin Rx by ATPIII; max IMT=1.2–3.5 mm HDL-C ≤1.6 mmol/l§	Aspirin 55%, beta blocker 24–29%; ACE inh or ARB 37–39%; no LLT ≥4 wks	2	2003–2006	*A*=0.83 *A*+*T*=0.83	*A*=1.3 *A*+*T*=1.32	101 (2.62)	48 (1.24)	167 (1.89)
[5]	
377
REGRESS⁎⁎	**P40**		131	56	0	CAD, symptomatic, coronary angiogram ≥50% reduction in ≥1 major coronary artery¶	No LLT ≤6 wks (≤12 wks for fibrates)	2	1989–1991	*P*=0.87 Pbo=0.86	*P*=1.08	168 (4.35)	38 (0.98)	163 (1.84)
Pbo=1.07
[8]	
Pbo		124
**Studies with statin arms**														
ENHANCE⁎	**S80**		342	46	51	hFH	Untreated, LDL-C >210 mg/dL 5.44 mmol/l), 81% prior statin use	2	2002–2006	*S*=0.68 *S*+*E*=0.68	*S*=0.8 *S*+*E*=0.8	319 (8.26)	47 (1.22)	159 (1.80)
[12]	S80+E10	

338
VYCTOR⁎⁎	**P40**		≥18	58	57	10 yr absolute risk for CHD or MI ≥20 per ATPIII	Low dose statins, none received E previously	1	2005	NA	*P*=1.33 *S*=1.3 *S*+*E*=1.23	130 (3.37)	45.3 (1.17)	192 (2.17)
[14]	**S40**	
S20+E10^b^		≥18
≥18
ARBITER II⁎	ERN 1000		87	67	9	Known CVD, LDL-C <130 mg/dL(<3.37 mmol/l) and HDL-C <45 mg/dL(<1.17 mmol/l)	All patients on statins (93% S)	1	2001–2003	ERN=0.89 Pbo=0.87	NA	89 (2.31)	40 (1.04)	163 (1.84)
[21]	**Pbo**		80

A=atorvastatin; ACE=angiotensin-converting enzyme; ARB=angiotensin-receptor blocker; CAD=coronary artery disease; CHD=coronary heart disease; CVD=cardiovascular disease;E=ezetimibe; hFH= heterozygous familial hypercholesterolemia L=lovastatin; Met=metropolo; P=pravastatin; Pac=pactimibe; Pbo=placebo; S=simvastatin; R=rosuvastatin; T=torcetrapib, W= warfarin.

⁎⁎⁎ Bold text denotes treatment arm in analysis.

⁎⁎ Study was statistically significant (*p*<0.05) for primary endpoint.

⁎ Study was non-significant (*p*>0.05) for primary endpoint.

† MI or hospitalization for unstable angina 3 mon–5 yr before enrollment and a total serum cholesterol 4–7 mmol/L (155–271 mg/dL) after 2 mon on low-fat diet; 88% men, 75% infarction, 5% diabetics.

‡ LDL-C 120–190 mg/dL (3.10–4.92 mmol/l) w/0–1 CHD risk factors or 120–160 mg/dL (3.10–4.14 mmol/l) w/≥2 CHD risk factors and 10 yr risk <10%, HDL-C ≤60 mg/dL (1.55 mmol/l); max IMT=1.2–3.5 mm.

§ LDL-C ≤160 - >220 mg/dL (4.1 mmol/L) w/10 yr CHD risk <10%; LDL‑C ≥130 -<190mg/dL (3.37–4.92 mmol/L) w/10 yr CHD risk ≥10% and ≤20%, LDL‑C ≥115 -<190 mg/dL (2.98–4.92 mmol/L) w/10 yr CHD risk >20%, and TG ≥150 - ≤500 mg/dL (1.70–5.65 mmol/L).

¶ Cholesterol 155–310 mg/dl (4.01–8.03 mmol/l).

a P20 initially, change to 40 mg based on LDL-C.

b S20+E10 initially, change to S40+E based on LDL-C.

**Table 2:** Pooled baseline mean maximum CCA/cIMT and annualized rates of change*.

	**Studies**	**Patients**	**Baseline mean max CCA/cIMT [mm]**†	**Annualized change mean max CCA/cIMT [mm/yr]**†‡	
	**#**	***N***	**Mean (SE)**	**Mean (SE)**	**95% CI**
Overall	12	5051	1.0866 (0.0032)	−0.0023 (0.0014)	(−0.0050, −0.0004)
Study year§					
Before 2000	5	1955	1.2550 (0.0059)	−0.0134 (0.0020)	(−0.0173, −0.0096)
After 2000	7	3096	1.0181 (0.0038)	0.0083 (0.0019)	(−0.0046, 0.0121)
CHD risk					
Yes	4	1242	1.3022 (0.0071)	0.0021 (0.0030)	(−0.0038, 0.0081)
No	8	3809	1.0317 (0.0036)	−0.0034 (0.0015)	(−0.0065, −0.0004)
hFH					
Yes	3	1677	0.9130 (0.0050)	0.0053 (0.0024)	(0.0005, 0.0100)
No	9	3374	1.2032 (0.0041)	−0.0059 (0.0017)	(−0.0091, −0.0026)
Thickened baseline IMT¶					
Yes	3	1964	1.1703 (0.0052)	−0.0135 (0.0020)	(−0.0175, −0.0095)
No	9	3087	1.0375 (0.0040)	0.0074 (0.0019)	(−0.0037, 0.0111)
LDL-C reduction					
<−27.6%	6	3259	1.1335 (0.0042)	−0.0036 (0.0016)	(−0.0066, −0.0005)
≥−27.6%	6	1792	1.0200 (0.0050)	0.0025 (0.0030)	(−0.0034, −0.0083)
Mean age (yr)					
<57	5	2237	0.9454 (0.0044)	−0.0045 (0.0017)	(−0.0077, −0.0012)
≥57	7	2814	1.2423 (0.0046)	0.0023 (0.0024)	(−0.0024, 0.0071)
Female (%)					
<40	5	1800	0.9853 (0.0058)	0.0204 (0.0043)	(0.0119, 0.0289)
≥40	7	3251	1.1299 (0.0038)	−0.0048 (0.0015)	(−0.0077, −0.0020)

hFH=heterozygous familial hypercholesterolemia.

* Studies included: ACAPS, ARBITER I, BCAPS, CAIUS, CAPTIVATE, ENHANCE simvastatin, REGRESS, METEOR, PLAC II, VYCTOR, and RADIANCE I & II (atorvastatin arms). The PLAC II study was excluded from the baseline analysis because of missing baseline standard error information.

† Pooled estimates for subgroups are weighted based on the inverse of the square of the standard error for the individual studies.

‡ SE and 95%CI are based on study-end values for the treatment difference (placebo-controlled) or change from baseline (monotherapy arms) in cIMT.

§ Studies conducted before 2000: ACAPS, BCAPS, CAIUS, PLAC II, REGRESS. Studies conducted after 2000: ARBITER I, CAPTIVATE, ENHANCE, METEOR, VYCTOR, and RADIANCE I & II (atorvastatinarms)..

¶ Entry criteria.

**Table 3:** Pooled baseline mean mean CCA/cIMT and annualized rates of change in studies before and after 2000 for subgroups.

	**Studies before 2000**⁎					**Studies after 2000**⁎⁎				
	**Studies**	**Patients**	**Baseline mean CCA/cIMT [mm]**†	**Annualized change mean CCA/cIMT [mm/yr]**†‡		**Studies**	**Patients**	**Baseline mean CCA/cIMT [mm]**	**Annualized change mean CCA/cIMT [mm/yr]**†‡	
**#**	***N***	**Mean (SE)**	**Mean (SE)**	**95% CI**	***#***	***N***	**Mean (SE)**	**Mean (SE)**	**95% CI**
CHD risk										
Yes	3	1201	0.8008 (0.0064)	−0.0184 (0.0051)	(−0.0283, −0.0084)	1	375	0.8300 (0.0072)	0.0080 (0.0020)	(0.0041, 0.0119)
No	2	1064	0.8898 (0.0054)	−0.0098 (0.0029)	(−0.0155, −0.0042)	7	3131	0.7367 (0.0024)	−0.0033 (0.0009)	(−0.0051, −0.0015)
hFH										
Yes	1	281	0.8645 (0.0094)	−0.0138 (0.0069)	(−0.0273, −0.0003)	3	1677	0.7435 (0.0036)	−0.0005 (0.0012)	(−0.0113, −0.0057)
No	4	1984	0.8492 (0.0046)	−0.0117 (0.0027)	(−0.0170, −0.0064)	5	1829	0.7473 (0.0029)	−0.0021 (0.0012)	(0.0003, 0.0043)
Thickened baseline cIMT§										
Yes	1	783	0.9026 (0.0067)	−0.0090 (0.0032)	(−0.0153, −0.0027)	4	1781	0.7782 (0.0036)	−0.0085 (0.0015)	(−0.0113, −0.0057)
No	4	1482	0.8207 (0.0053)	−0.0167 (0.0041)	(−0.0247, −0.0087)	4	1725	0.7242 (0.0029)	0.0023 (0.0010)	(0.0003, 0.0043)
LDL-C reduction (median)										
<−27.6%	2	1305	0.8484 (0.0047)	−0.0096 (0.0030)	(−0.0156, −0.0036)	4	1781	0.7782 (0.0036)	0.0021 (0.0011)	(0.0000, 0.0042)
≥−27.6%	3	960	0.8645 (0.0086)	−0.0170 (0.0045)	(−0.0258, −0.0082)	4	1725	0.7242 (0.0029)	−0.0066 (0.0013)	(−0.0092, −0.0040)
Mean age (yr)										
<57	2	536	0.8645 (0.0086)	−0.0148 (0.0066)	(−0.0277, −0.0019)	3	1677	0.7435 (0.0036)	−0.0005 (0.0012)	(−0.0028, 0.0019)
≥57	3	1729	0.8484 (0.0047)	−0.0115 (0.0027)	(−0.0168, −0.0061)	5	1829	0.7473 (0.0029)	−0.0021 (0.0012)	(−0.0043, 0.0002)
Female (%)										
<40	3	1201	0.8008 (0.0064)	−0.0184 (0.0051)	(−0.0283, −0.0084)	4	1465	0.7835 (0.0040)	0.0094 (0.0019)	(0.0057, 0.0130)
≥40	2	1064	0.8898 (0.0054)	−0.0098 (0.0029)	(−0.0155, −0.0042)	4	2041	0.7277 (0.0027)	−0.0040 (0.0009)	(−0.0058, −0.0021)

hFH=heterozygous familial hypercholesterolemia; CHD=coronary heart disease; IMT=statins.

⁎ Studies before 2000 included REGRESS, ASAP, LIPID, and BCAPS, KAPS study was excluded from the baseline analysis due to missing baseline standard error information.

⁎⁎ Studies after 2000 included ARBITER I, METEOR, CASHMERE, CAPTIVATE, ENHANCE (simvastatin arm), ARBITER II (placebo arm), and Radiance I & II (atorvastatin arm).

† Pooled estimates for subgroups are weighted based on the inverse of the square of the standard error for the individual studies.

‡ SE and 95%CI are based on study-end values for the treatment difference (placebo-controlled) or change from baseline (monotherapy arms) in cIMT.

§ Entry criteria.

**Table 4:** Pooled baseline mean maximum CCA/cIMT and annualized rates of change in studies before and after 2000* for subgroups.

	Studies before 2000*					**Studies after 2000**⁎⁎				
	**Studies**	**Patients**	**Baseline max CCA/cIMT [mm]**†	**Annualized change max CCA/cIMT [mm/yr]**†‡		**Studies**	**Patients**	**Baseline max CCA/cIMT [mm]**	**Annualized change max CCA/cIMT [mm/yr]**†‡	
**#**	***N***	**Mean (SE)**	**Mean (SE)**	**95% CI**	***#***	***N***	**Mean (SE)**	**Mean (SE)**	**95% CI**
CHD risk										
Yes	3	867	1.3028 (0.0080)	−0.0140 (0.0038)	(−0.0215, −0.0066)	1	375	1.3000 (0.0150)	0.0300 (0.0050)	(0.0202, 0.0398)
No	2	1088	1.1975 (0.0088)	−0.0132 (0.0023)	(−0.0177, −0.0087)	6	2721	0.9989 (0.0039)	0.0046 (0.0021)	(0.0005, 0.0087)
hFH										
Yes	0	0	–	–	–	3	1677	0.9130 (0.0050)	0.0053 (0.0024)	(0.0005, 0.0100)
No	5	1955	1.2550 (0.0059)	−0.0134 (0.0020)	(−0.0173, −0.0096)	4	1419	1.1547 (0.0057)	0.0135 (0.0032)	(0.0074, 0.0197)
Thickened baseline cIMT§										
Yes	2	1088	1.1975 (0.0088)	−0.0132 (0.0023)	(−0.0177, −0.0087)	1	876	1.1553 (0.0065)	−0.0145 (0.0042)	(−0.0228, −0.0062)
No	3	867	1.3028 (0.0080)	−0.0140 (0.0038)	(−0.0215, −0.0066)	6	2220	0.9486 (0.0046)	0.0143 (0.0022)	(0.0101, 0.0186)
LDL-C reduction (median)										
<−27.6%	3	1549	1.2642 (0.0061)	−0.0136 (0.0020)	(0.0176, −0.0097)	3	1710	1.0180 (0.0057)	0.0112 (0.0024)	(0.0064, 0.0160)
≥−27.6%	2	406	1.0747 (0.0269)	−0.0088 (0.0095)	(−0.0275, 0.0099)	4	1386	1.0181 (0.0050)	0.0037 (0.0031)	(−0.0025, 0.0098)
Mean age (yr)										
<57	2	560	1.0515 (0.0091)	−0.0132 (0.0023)	(−0.0177, −0.0087)	3	1677	0.9130 (0.0050)	0.0053 (0.0024)	(0.0005, 0.0100)
≥57	3	1395	1.4045 (0.0078)	−0.0139 (0.0038)	(−0.0214, −0.0065)	4	1419	1.1547 (0.0057)	0.0135 (0.0031)	(0.0074, 0.0197)
Female (%)										
<40	2	406	1.0747 (0.0269)	−0.0088 (0.0095)	(−0.0275, 0.0099)	3	1394	0.9809 (0.0060)	0.0280 (0.0049)	(0.0185, 0.0375)
≥40	3	1549	1.2642 (0.0061)	−0.0136 (0.0020)	(−0.0176, −0.0097)	4	1702	1.0430 (0.0049)	0.0047 (0.0021)	(0.0006, 0.0088)

hFH=heterozygous familial hypercholesterolemia; CHD=coronary heart disease; cIMT=carotid intima media thickness.

* Studies conducted before 2000: ACAPS, BCAPS, CAIUS, PLAC II, REGRESS.

** Studies conducted after 2000: ARBITER I, CAPTIVATE, ENHANCE, METEOR, VYCTOR, and RADIANCE I & II (atorvastatin arms).

† Pooled estimates for subgroups are weighted based on the inverse of the square of the standard error for the individual studies.

‡ SE and 95%CI are based on study-end values for the treatment difference (placebo-controlled) or change from baseline (monotherapy arms) in cIMT.

§ Entry criteria.
